# MicroRNA-223 demonstrated experimentally in exosome-like vesicles is associated with decreased risk of persistent pain after lumbar disc herniation

**DOI:** 10.1186/s12967-017-1194-8

**Published:** 2017-05-01

**Authors:** Aurora Moen, Daniel Jacobsen, Santosh Phuyal, Anna Legfeldt, Fred Haugen, Cecilie Røe, Johannes Gjerstad

**Affiliations:** 1 National Institute of Occupational Health, Pb 8149 Dep., 0033 Oslo, Norway; 2 Department of Physical Medicine and Rehabilitation, Oslo University Hospital, Oslo, Norway; 3 Faculty of Medicine, University of Oslo, Oslo, Norway; 4 Department of Biosciences, University of Oslo, Oslo, Norway

**Keywords:** Lumbar radicular pain, Disc herniation, Inflammation, Immune response, microRNA-223, miR-223

## Abstract

**Background:**

Previous findings have demonstrated that lumbar radicular pain after disc herniation may be associated with up-regulation of inflammatory mediators. In the present study we examined the possible role of extracellular microRNAs (miRs) in this process.

**Methods:**

Single unit recordings, isolation of exosome-like vesicles, electron microscopy, nanoparticle tracking analysis, western blot analysis and qPCR were used in rats to demonstrate the effect of nucleus pulposus (NP) applied onto the dorsal nerve roots. ELISA and qPCR were used to measure the level of circulating IL-6 and miRs in a 1-year observational study in patients after disc herniation.

**Results:**

In the rats, enhanced spinal cord nociceptive responses were displayed after NP applied onto the dorsal nerve roots. An increased release of small non-coding RNAs, including miR-223, miR-760 and miR-145, from NP in exosome-like vesicles was demonstrated. In particular, the NP expression of miR-223, which inhibited the nociceptive spinal signalling, was increased. In the patients, increased extracellular miR-223 was also verified in the acute phase after disc herniation. The increased miR-223 expression was, however, only observed in those who recovered (sex, age and smoking were included as covariates).

**Conclusions:**

Our findings suggest that miR-223, which can be released from the NP after disc herniation, attenuates the neuronal activity in the pain pathways. Dysregulation of miR-223 may predict chronic lumbar radicular pain.

*Trial registration/ethics* REK 2014/1725

**Electronic supplementary material:**

The online version of this article (doi:10.1186/s12967-017-1194-8) contains supplementary material, which is available to authorized users.

## Background

Experimental data suggest that lumbar disc herniation may induce sensitization of the primary afferent nerve fibers, even in the absence of nerve root compression [1–5]. The local release of inflammatory molecules, such as interleukin-6 (IL-6), nitric oxide (NO), prostaglandin E2 (PGE2) and matrix metalloproteinases (MMPs) [6], may be a crucial part of this process. For instance, the interaction between the nucleus pulposus (NP) cells and recruited immune cells may up-regulate IL-6 and MMPs [7, 8]. The circulating cytokine levels are also elevated in patients with moderate or severe pain following disc herniation [9, 10].

Such local or systemic inflammatory processes can be modulated by specific intracellular microRNAs (miRs), i.e., small non-coding RNAs that act by binding to the 3’UTR (untranslated region) of target mRNA, resulting in either translational repression or mRNA degradation [11]. Previous data suggest that miR-145 may regulate immune cells [12], and that inflammatory responses in the intervertebral disc may be reduced by miR-146a [13]. Moreover, decreased miR-155 and increased miR-27a in the disc may be associated with apoptosis [14, 15], whereas up-regulation of miR-10b and miR-21 is linked to NP cell proliferation [16, 17].

Interestingly, biologically active miR-223 [18] can be exchanged between cells via exosomes, which are small extracellular micro-vesicles that contain RNA and protein cargos [19]. Therefore, miRs released in exosomes may affect intracellular signalling in adjacent cells [20, 21]. Some extracellular miRs can also directly interact with cell-surface receptors [22]. For example, miR-let-7b may directly activate nociceptive nerve fibers and elicit pain via TLR7 and TRPA1 [23]. Additionally, extracellular miRs are present in human serum and plasma, where they appear to be resistant to RNAase degradation. A number of protein-encoding genes may be regulated by miRs [24].

In patients, down-regulated circulating miR expression has been reported in migraines [25], complex regional pain syndrome [26], fibromyalgia [27] and rheumatoid arthritis [28]. Hence, we hypothesized that miRs could be involved in inflammatory processes and influence the risk of persistent pain after disc herniation. The present study suggests that miR-223 may be linked to the recovery rate in lumbar radicular pain patients.

## Methods

### Animals and surgery

Inbred female Lewis rats were used in the experiments. The animals were sedated with isoflurane gas and anesthetized with urethane (~2 g/kg bodyweight, i.p.). Absence of paw withdrawal and ear reflex to pinch indicated adequate surgical anaesthesia. The rat core temperature was maintained at 36–37 °C with a feedback heating pad. A laminectomy, with a 1-mm lateral expansion on the left side for applying NP, was performed at vertebrae Th13-L1, corresponding to spinal cord segments L3–S1. The vertebral column was rigidly fixed by clamps rostral and caudal to the exposed spinal cord segments. The meninges, i.e., dura mater and arachnoidea, were punctured by a cannula and carefully removed by two tweezers. A section of 8–10 mm of the sciatic nerve was freed at the mid-thigh level and isolated from the surrounding tissue by a plastic film. A caudectomy was performed on genetically identically donor rats, and NP was harvested from 3 to 8 caudal intervertebral discs.

All animal experiments were approved by the Norwegian Animal Research Authority (NARA) and were performed in conformity with the laws and regulations controlling experiments and procedures on live animals in Norway. The rats were euthanized immediately after the end of the experiments.

### Electrophysiological recordings

A bipolar silver hook electrode (1.5 mm between the hooks) was placed proximal to the main branches of the sciatic nerve for electrical stimulation. A parylene-coated tungsten microelectrode with impedance of 2–4 MΩ (Frederick Haer & Co, Bowdoinham, USA) was lowered into the left dorsal horn by an electrically controlled micromanipulator (Märzhäuser Wetzlar GmbH & Co. KG, Wetzlar, Germany), and a reference electrode was placed subcutaneously. Extracellular single cell recordings were performed at depths of 100–600 µm from the surface of the spinal cord [29]. Only one cell was studied in each animal. The recorded signals were amplified, bandpass filtered with a half amplitude cut of 500–1250 Hz (Digitimer Ltd, Hertfordshire, UK), digitalized using a CED 1401 μ interface and displayed on a computer screen by means of the CED Spike 2.2 software (Cambridge Electronic design, Cambridge, UK). The sampling frequency was 20 kHz.

The A- and C-fiber responses were separated according to latencies, where spikes 50–300 ms after stimulus were defined as C-fiber responses. Single cell recordings were ensured by the amplitude and shape of the action potentials. Every 4th min a single test stimulus (2 ms rectangular pulse, 1.5 × C-fiber threshold) was applied to the sciatic nerve through the hook electrode. A pulse buffer connected to a stimulus isolator unit (NeuroLog System, Digitimer Ltd, Hertfordshire, UK) controlled the stimulus intensity. The C-fiber threshold was defined at the beginning of each experiment as the lowest stimulus intensity necessary to evoke a C-fiber response. Six stable C-fiber responses, varying less than 20%, served as a baseline for the subsequent experiments. Cells were rejected if the C-fiber response consisted of fewer than five or more than 20 spikes at the start of the recording.

Nucleus pulposus was harvested from 3 to 8 caudal vertebrae of genetically identical donor rats and carefully applied caudally to the recording electrode, covering the incoming spinal dorsal nerve roots [30]. The C-fiber responses were followed for 180 min after application of NP transplant covering 1–2 mm of the dorsal nerve roots, or 40 µL 0.6 mg/mL miR-223-3p in Invivofectamine (Invitrogen, Carlfbad, USA). In accordance with our earlier studies [30, 31], NP was applied in absence of nerve root compression. Application of 40 µL 0,9% NaCl served as controls.

### mRNA analysis

As previously described [30], total RNA (RIN values were >7) was isolated from frozen NP tissue and converted into cDNA. The qPCR was performed in two parallels on a StepOnePlus qPCR machine (Applied Biosciences, USA). IL-6 primers (FW; TGCCCTTCAGGAACA; RV; AAGGCAGTGGCTGTC) were designed using Primer Express 2.0 (Applied Biosystems, California, USA) and checked for specificity by performing a BLAST search. Effort was made to design primers without non-specific binding (the melting curves indicated no bi-products). Target genes were normalized to β-actin (FW; CTAAG GCCAA CCGTG AAAAG A, RV; ACAAC ACAGC CTGGA TGGCT A) (internal reference).

### miR analysis

After transcardial perfusion with HBSS, NP tissue was harvested from 3 to 8 caudal vertebrae from the same donor rat and then bisected. One piece was immediately frozen after caudectomy (NP^nativ^), and the other piece was applied onto the spinal dorsal nerve roots for 180 min before it was frozen (NP^exposed^). For the NP graft in media, the tail of a donor rat was cut off at the base, skinned and placed in a sterile petri dish. The NP tissue was harvested from the caudal vertebrae in a LAF bench. The tissue was incubated in 0.5 mL of medium in a humidified 5% CO_2_ incubator at 37 °C. Two groups of samples were provided, differing in the time spent in the incubator—5 min and 3 h. After incubation, the samples were spun first at 300*g* and then at 1000*g* in order to retain medium without any cells. miR extraction was performed on 200 µL of medium. In accordance with the manufacturer’s protocol, total RNA was isolated from frozen (−80 °C) tissue or 200 µL of medium using the miRNeasy micro kit (Qiagen) and converted into cDNA using the miScript HiSpec reverse transcription buffer (Qiagen).

### qPCR array

The screening of miRs was performed using the Rat Pain: Neuropathic and Inflammatory MiScript miR PCR Array containing 84 miRs (cat.no. MIRN-120Z, Qiagen). cDNA from NP^nativ^ and NP^exposed^ were separately pooled before the analyses. Normalization was performed to the mean of all six snoRNA/snRNAs controls included in each array.

Based on the array analysis, miRs with a fold change >5 were selected for follow-up analysis with qPCR. In this case, the mean of the SNORD61 and SNORD68 reference genes was used for normalization of gene expression. All primers were predesigned and delivered by Qiagen. Then, 1 ng of cDNA was used in the qPCR reaction. qPCR was performed on an Applied Biosystems 7900 Real Time PCR System with the following conditions: 95 °C for 15 min, followed by 40 cycles at 95 °C for 5 s, 55 °C for 30 s, and 70 °C for 30 s.

### Exosome-like vesicle (ELV) isolation

Nucleus pulposus grafts were incubated in the serum-free culture media (Ham’s F-12 nutrient mixture) at 37 °C in a humidified 5% CO_2_ incubator for 3 h. After incubation, media were centrifuged at 300*g* for 10 min and then at 1000*g* for 10 min to remove cells and other larger contaminants. Media were then centrifuged for 45-min at 10,000*g*. Pellets were discarded and supernatants were passed through a 0.45-µm filter. Supernatants were ultracentrifuged (Optima MAX-XP Benchtop Ultracentrifuge, Beckman Coulter, Brea, CA, USA) at 100,000*g* for 90 min to pellet ELVs. The pellet was washed once with PBS and centrifuged again at 100,000*g* for another 90 min. The ELV pellets were resuspended in PBS and stored at −21 °C for further analyses. As previously described [32], all centrifugation steps were performed at 4 °C.

### Electron microscopy

The ELVs pellets resuspended in PBS were fixed in 4% formaldehyde/0.2% glutaraldehyde and placed on formvar-carbon-coated electron microscopy grids. The grids were then washed, and stained with a mixture of methylcellulose and uranylacetate. Samples were observed in a JEOL–JEM 1230 (JEOL Ltd., Tokyo, Japan) at 80 kV and pictures were acquired using a Morada camera and iTEM software (Olympus, Münster, Germany).

### Nanoparticle tracking analysis (NTA)

Nanoparticle tracking analysis was used to determine the size distribution and concentration of the ELV population. Exosome pellets were resuspended in PBS, vortexed thoroughly for 1 min and loaded into the NS300 instrument (Malvern Instruments, Worcestershire, UK) using the syringe pump. For all measurements, five videos, 60 s each, were acquired (infusion rate: 20 with camera settings: shutter, 600; gain, 350–450). Recorded data were processed with the NTA 3.0 software to obtain the size distribution and approximate concentration by tracking the centre of each particle under Brownian motion on a frame-by-frame basis.

### SDS-PAGE and immunoblotting

Exosome-like vesicles were lysed in lysis buffer containing 50 mM Tris–HCl, 300 mM NaCl, 1 mM EDTA, 0.5% Triton X-100 (pH 7.4) in the presence of Halt™ protease inhibitor cocktail (ThermoFisher Scientific, Waltham, MA, USA). ELV proteins were separated by SDS-PAGE in 4–20% TGX™ precast gels (BioRad, Hercules, CA, USA) after solubilization in laemmli sample buffer. Proteins were transferred to PVDF membranes using a Tranfer-Blot Turbo Transfer Pack (BioRad). Membranes were blocked in 5% (w/v) non-fat dry milk dissolved in PBS containing 0.1% (v/v) Tween-20. Blots were incubated with the specified primary and secondary antibodies. Blots were visualized with Amersham™ ECL™ Prime Western blot detection (GE Healthcare, Little Chalfont, UK) on an Amersham AI600RGB imaging instrument (GE Healthcare).

### Antibodies

The antibodies used for western blot analysis were as follows: mouse anti-tsg101 (Santa Cruz Biotechnology Inc., Dallas, TX, USA); rabbit anti-CD9 (Abcam, Cambridge, UK); rabbit anti-alix (Merck Millipore, Darmstadt, Germany); and HRP-conjugated secondary antibodies (Cell Signalling Technology, Danvers, MA, USA). All antibody dilutions (1:500 for the primary and 1:5000 for the secondary antibodies, respectively) were prepared in blocking solution.

### Patients

Participants with lumbar radicular pain were recruited from Oslo University Hospital (Ullevål) during 2007–2009. A total of 122 patients were included in the intended follow-up assessment, 8% were lost during the follow-up, and 112 patients were ultimately assessed at the 12-month follow-up. All participants received written information and signed an informed consent form. The study was approved by the Norwegian Social Science Data Services and Norwegian Regional Committee for Medical Research Ethics (REK 214/1725).

The inclusion criteria were patients aged 18–60 years, LDH on magnetic resonance imaging with corresponding radicular pain, and positive straight leg raise test results. A positive straight leg raise test was defined by pain radiating into one or both legs when the examiner slowly raised the straightened limb until 60°. The test was performed in the supine position and supplemented with slight dorsiflexion of the foot. The exclusion criteria were lumbar spinal stenosis, previous spinal surgery for a herniated disc at the same level (to rule out radiating pain from epidural fibrosis), fusion surgery at any level in the lumbar spine, generalized musculoskeletal pain (widespread musculoskeletal pain in three out of four body quadrants), inflammatory rheumatic disease, diabetic polyneuropathy, cardiovascular disease (New York Heart Association III and IV), cancer, psychiatric disease, drug abuse and alcoholism, recent surgery (within 1 month), pregnancy, poor proficiency in the Norwegian language, and non-European-Caucasian ethnicity. Patients with cauda equina syndrome were excluded.

### Clinical procedure

At the initial screening visit and 1-year follow-up, the patients underwent a standardized clinical examination, which included an assessment of sensory and motor function, and standardized registrations of pain and function. Pain intensity was recorded using the visual analogue scale (VAS) with anchor values from 0 (no pain) to 10 (worst imaginable pain) over the past week during activity.

Demographic data and the patient’s smoking status were obtained upon inclusion in the study. Treatment (surgery or conservative) was determined during the initial screening visit, where surgical treatment was given to patients with persistent radicular pain lasting for more than 8 weeks, neurological deficits (sensory changes, muscle weakness or depressed or absent deep tendon reflexes) and corresponding magnetic resonance imaging findings in the anticipated location. Non-surgical treatment was given to patients who did not clearly meet these criteria. Non-surgical treatment included a brief cognitive intervention, activity guidance during the acute phase of lumbar radicular pain, and physiotherapy for most patients.

### Blood sampling and RNA isolation

At inclusion and 12-month follow-up, venous blood was collected and kept on ice for 45 min. After centrifugation at 2000*g* and 4 °C and for 10 min, the supernatant serum was collected and stored in aliquots at −80 °C until further analysis. Serum was used to analyse the miRs. Samples with visible haemolysis were excluded. Total small RNAs were extracted from 200 µL serum using the miRNeasy serum plasma isolation kit (Qiagen) according to the manufacturer’s protocol. Synthetic C-elegans (C) miR-39-3p (Qiagen) was spiked-in at a final concentration of 1.6 × 10^8^ copies/μL after the initial denaturation, prior to extraction, to correct the extraction efficiency. Total RNA was eluted in 14 µL of RNase-free water. A fixed volume of 7 µL of eluate was used as input for the cDNA synthesis. RNA was converted to cDNA using the qScript microRNA cDNA synthesis kit (Quanta). qPCR was performed with PerfeCta SYBR green supermix on an Applied Biosystems 7900 Real Time PCR System with the following conditions: 95 °C for 2 min, followed by 40 cycles at 95 °C for 5 s, 60 °C for 15 s, and 70 °C for 15 s. Data were normalized to the spiked-in C miR-39-3p.

Of the 112 patients with both baseline and 12-months data, 7 and 3 samples were excluded due to visual haemolysis at inclusion and 12 months, respectively. In addition, 1 sample was missing at inclusion, 2 samples were missing at 12 months and 2 samples could not be analysed due to undetectable miR levels at 12 months. In total, this resulted in miR data for both inclusion and 12 months from 97 patients.

### Measurements of the cytokine concentration in serum by ELISA-methodology

As previously described [9], IL-6 concentrations in the serum of the same 97 patients were determined by using commercial ELISA kits (Human ultra-sensitive kits for IL-6, Invitrogen Corporation, CA, USA). Following the manufacturer’s instructions, 100 µL of the serum samples or standards were added to each well in microtiter plates that were pre-coated with an antibody specific to IL-6 and incubated at room temperature. Serum collected at the two time points for each patient was analysed on the same microtiter plate.

### Statistics

The spinal nociceptive activity, i.e., the C-fiber response, was presented as percent of baseline. Baseline recordings were converted to an average of three consecutive responses, whereas the recordings after NP application were converted to an average of nine consecutive responses, producing two pre-NP/miR-233-3p and five post-NP/miR-233-3p values. The effect of NP or miR-233-3p on spinal nociceptive response over time was compared to the control by repeated measures analysis of variance (rmANOVA). When the sphericity assumption was not met, a Greenhouse–Geisser correction was applied.

For miR arrays, data analysis was performed using the miScript miR PCR Array Web-based software delivered by Qiagen (http://pcrdataanalysis.sabiosciences.com/mirna). In brief, Δ *C*_t_value for each miR profiled in the plate is calculated using the formula Δ*C*_t_ = *C*_t_^miRNA^ – AVG *C*_t_^SN1/2/3/4/5/6^, where AVG *C*_t_^SN1/2/3/4/5/6^ is the mean of all six snoRNA/snRNAs controls included on each array. ΔΔ*C*_t_ for each miR across the two groups of samples is calculated using the formula: ΔΔ*C*_t_ = Δ*C*_t_ (sample) −Δ*C*_t_ (control). The fold-change was calculated as 2^−ΔΔ*C*t^.

In the qPCR analysis, fold change values for each sample were defined by the expression of the target gene normalized to the expression of the mean of the reference genes SNORD61 and SNORD68 in the animal studies and C miR-39-3p in the patients. The abundance of each miR was calculated by the comparative Ct method and 2^−ΔCt^ values in animals were analysed by the two-tailed unpaired Student’s t test and linear mixed model.

Regarding the clinical arm of the study, the change in IL-6 and miRs from inclusion to 12 months was analysed by the paired Student’s t test. A two-sided Pearson correlation test was performed to examine the relationship between miR-223-3p at inclusion and IL-6 at 12 months and the delta miR values versus delta VAS values. In addition, the miR levels were analyzed by linear regression adjusted for sex, age and smoking. Baseline differences were tested by Pearson Chi square, unpaired Student’s t test and Mann–Whitney U test.

## Results

**Fig. 1:**
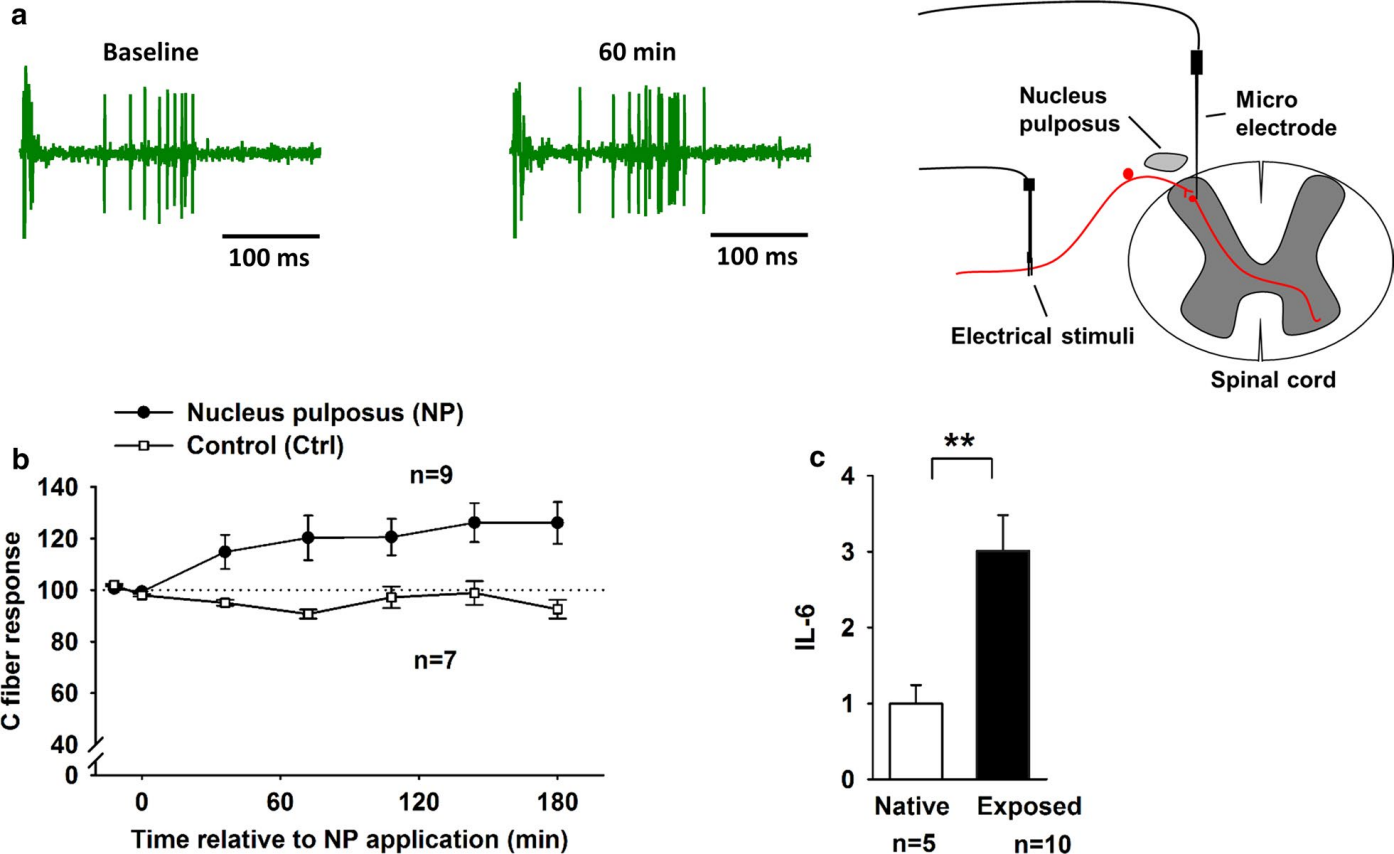
**a** Example of the of electrically evoked C-fiber responses in the dorsal horn neurons at baseline i.e., before and 180 min after the application of nucleus pulposus (NP) onto the dorsal nerve roots. **b** The time course of electrically evoked C-fiber responses expressed as the percent of baseline following the application of NP (*black*) and control (Ctrl, *white*); within subjects effects rmANOVA, p = 0.003. **c** Fold expression of IL-6 in NP tissue frozen directly (native) or 180 min after NP application onto the dorsal nerve roots (exposed) after harvesting. Students t test **p < 0.01. Data are given as the mean ± SEM

Application of NP onto the dorsal nerve-roots increased the evoked spinal C-fiber responses (Fig. 1a). Most cells reached a plateau within 180 min. However, the C-fiber response in the control group remained at baseline level (Fig. 1b). Over time, a significant increase in the C-fiber response was observed in the NP group (within subject rmANOVA, p = 0.003, NP group vs control group). A significant increase in the NP expression of IL-6 was also observed at 180 min in the exposed NP tissue compared to native tissue (Fig. 1c).

**Fig. 2:**
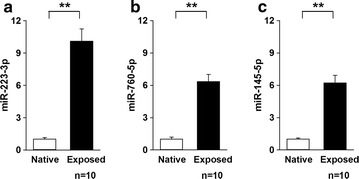
Fold expression of **a** miR-223, **b** miR-760 and **c** miR-145 in nucleus pulposus (NP) tissue frozen directly (native) or 180 min after NP application onto the dorsal nerve roots (exposed) after harvesting. Students t test **p < 1 × 10^−5^. Data are given as the mean ± SEM

Analyses of the pooled NP samples using the PCR array demonstrated a more than fivefold increase in the expression of 8 of the 84 miRs in NP following contact with the dorsal nerve roots (Additional file 1: Table S1). Validation with qPCR suggested that 6 of these miRs were more than fivefold up-regulated in exposed NP tissue compared to native NP tissue (Table 1). Interestingly, miR-223-3p, miR-760-5p and miR-145-5p, were also significantly up-regulated after correction for multiple testing (Fig. 2).

**Table 1:** miRs more than fivefold up-regulated in exposed NP tissue

miRs	Fold change	*p* value
206-3p	16.87 (5.92)	0.032
223-3p	10.10 (1.14)	3.1 × 10^−5^ **
451-5p	7.43 (1.63)	0.005
760-5p	6.34 (0.67)	2.3 × 10^−5^ **
145-5p	6.20 (0.73)	7.6 × 10^−5^ **
142-3p	5.95 (1.28)	0.005

Asterisks represent level of significance after conservative correction (Bonferroni, n = 84) for multiple testing. ** p < 0.0001

**Fig. 3:**
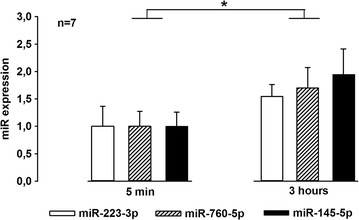
Fold expression of **a** miR-223, **b** miR-760 and **c** miR-145 in cell-free medium frozen after nucleus pulposus (NP) incubation for 5 min or 3 h. Overall linear mixed model, beta = 0.65, **p < 0.001, 95% CI (0.28, 1.01)

To further investigate whether these miRs could be actively released from NP cells, NP was isolated and incubated in vitro (without contact with the nerve). After 5 min and 3 h of incubation the cells were spun down, and the media were analysed by qPCR. miR-223-3p, miR760-5p and miR-145-5p were also found in the media fraction (Fig. 3). A significant overall increase in the miRs from 5 min to 3 h was observed [linear mixed model, beta = 0.65, p = 0.001, 95% CI (0.28, 1.01)].

**Fig. 4:**
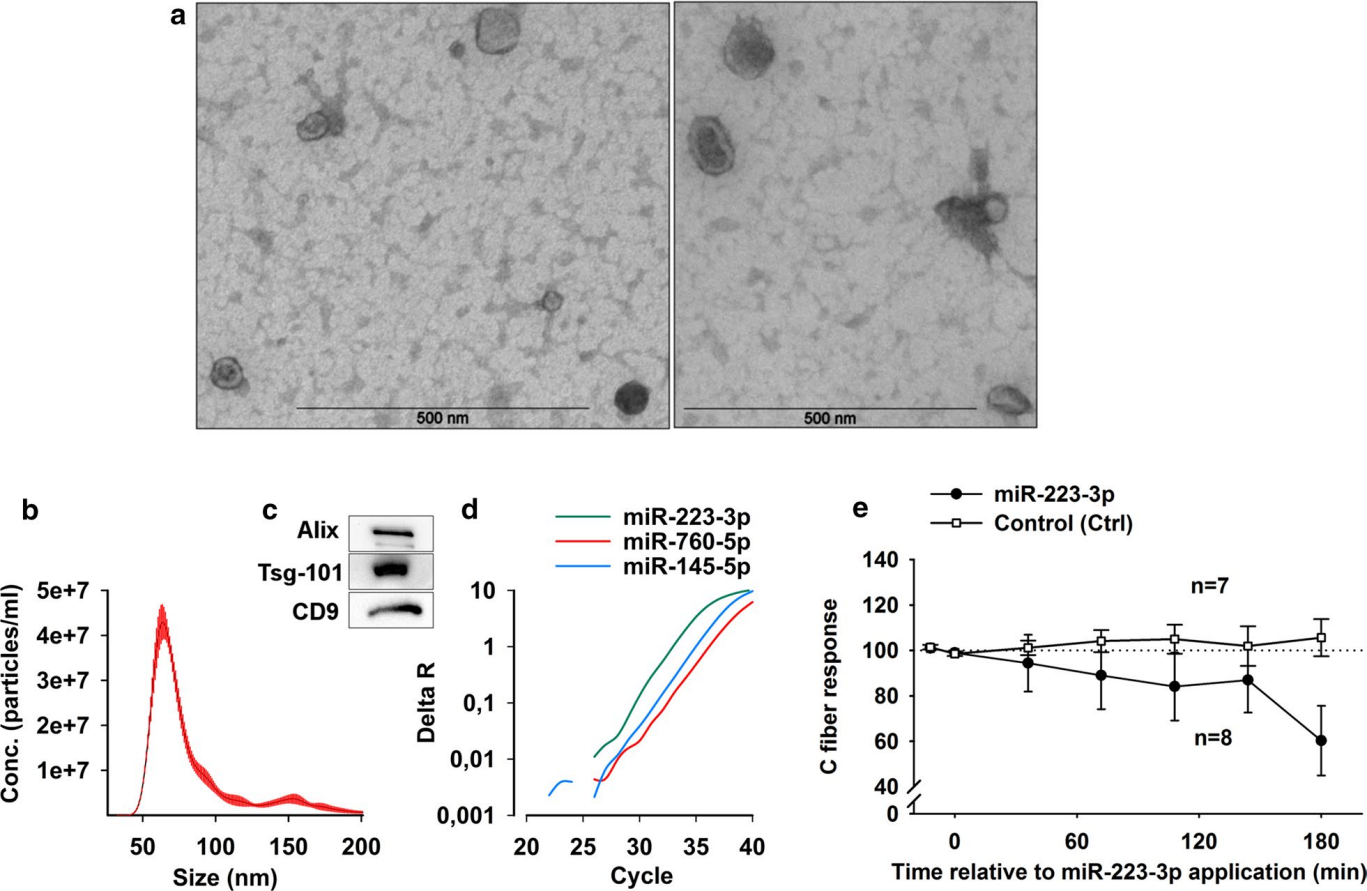
**a** Identification of exosome-like vesicles (ELVs). The morphology of the ELVs was inspected by electron microscopy. NP was excised from the tail of rats and incubated in serum-free media prior to isolation of ELVs by ultracentrifugation. **b** The size distribution of the ELV population was measured by NTA. Error bars are shown in *red*. **c** Several exosome-associated proteins were detected in the ELV fraction by western blot. Representative western blot showing alix, tsg101 and CD-9. **d** Examples of miR-223, miR-760 and miR-145 qPCR amplification plot in the exosome fraction. **e** Electrically evoked C-fiber responses in the dorsal horn neurons at baseline i.e., before and 180 min after the application of miR-223 onto the dorsal nerve roots; within subjects effects rmANOVA, p = 0.037. Data are given as the mean ± SEM

Electron microscopy, nanoparticle tracking analysis, western blot analysis and qPCR were used to demonstrate exosome-like vesicles (ELV) in the media conditioned for 3 h by NP. The ELVs population in the media appeared as homogenous single vesicles (Fig. 4a). In line with this observation, size distribution analysis with Nanosight (nanoparticle tracking analysis) revealed that most ELVs were between 50 and 100 nm in diameter, with an estimated mode size of 65 ± 1.4 nm (Fig. 4b). The average diameter of the ELVs was estimated to be 88.5 ± 0.7 nm, thus indicating presence of aggregates and/or larger vesicles.

Moreover, to verify the exosome-like vesicular nature of the obtained pellet, samples were subjected to western blot and blots were probed for specific proteins that are commonly associated with exosomes (Fig. 4c). We detected the presence of alix, tsg-101 and CD9 in our ELV preparations. Additional qPCR analysis of the ELV samples showed that miR-223-3p, miR760-5p and miR-145-5p were present in the exosome fraction (Fig. 4d). Application of miR-223-3p onto the spinal nerve roots decreased the C-fiber response in the dorsal horn neurones within 180 min (Fig. 4e).

**Fig. 5:**
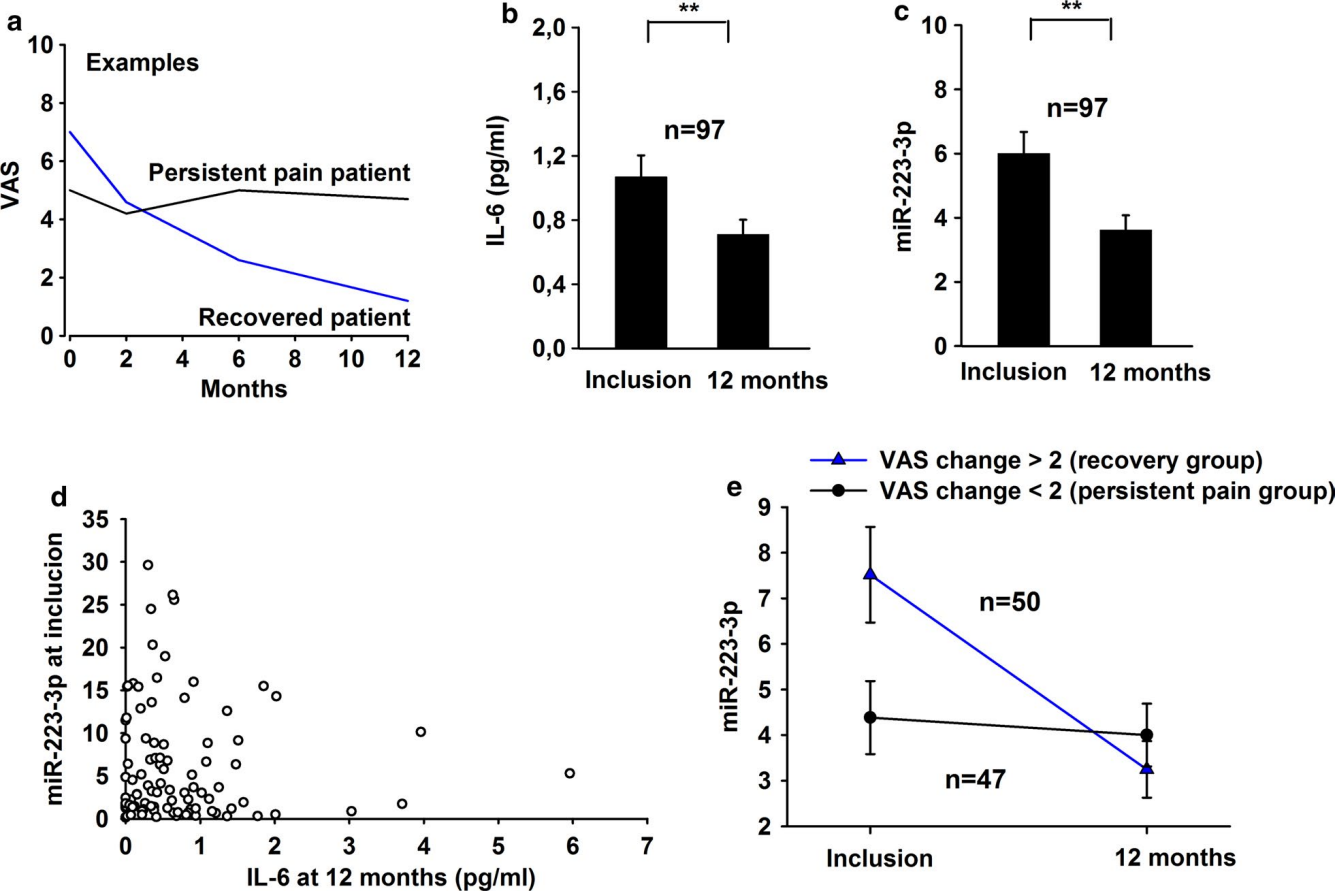
**a** Typical examples of the VAS scores of two patients after disc herniation. **b**, **c** IL-6 and miR-223 levels in the serum of patients with lumbar radicular pain at inclusion and 12 months. Paired Students t test, **p < 0.01. **d** miR-223 at inclusion versus IL-6 at 12 months in the serum of patients with lumbar radicular pain. **e** miR-223 levels at inclusion in the recovery group versus the persistent pain group defined as reduction in VAS from inclusion to 12 months. Linear regression adjusted for the sex, age and smoking, [beta = −2.97, p = 0.031, 95% CI (−5.67, −0.27)]

Not all patients recovered (Fig. 5a). The serum analyses in patients demonstrated a significant decrease in IL-6 (Fig. 5b) and miR-223-3p (Fig. 5c) from inclusion to 12 months (paired Student’s t test, p = 0.005), which was not the case for miR-760-5p and miR-145-5p (data not shown). No significant association between miR-223-3p at inclusion versus IL-6 at 12 months (Fig. 5d) was demonstrated (Pearson’s correlation; rho = −0.04, p > 0.05).

Further analyses indicated that the change in the miR-223-3p expression (Additional file 2: Figure S1) from inclusion to 12 months were correlated with the change in pain (delta miR values versus delta VAS, two-sided Pearson correlation, r = 0.221, p = 0.029).

For the final analyses the patients were grouped into recovery and persistent pain groups defined by reduction in VAS score from inclusion to 12 months; recovery group VAS change >2 and persistent pain group VAS change <2 (Fig. 5e). Differences in the sex, age, treatment and smoking (Table 2) were included as covariates in the analyses. Significantly higher miR-223-3p levels at inclusion were observed in the recovery group than in the persistent pain group [beta = −2.97, p = 0.031, 95% CI (−5.67, −0.27)].

**Table 2:** Characteristics of patients grouped in recovery and persistent pain group

	Recovery group n = 50	Persistent pain group n = 47	*p* value
Gender, men/women (%)	22/28 (44/56)	18/29 (38/62)	n.s^a^
Mean age (min–max)	40 (22–58)	42 (21–59)	n.s^b^
Current smoker, yes/no (%)	15/35 (30/70)	19/28 (40/60)	n.s^a^
Treatment, surgery/conservative (%)	23/27 (46/54)	13/34 (28/72)	<0.05^a^

The recovery group was defined by a reduction in VAS (VAS inclusion—VAS 12 months) >2.0, whereas the persistent pain group was defined by a reduction in VAS (VAS inclusion—VAS 12 months) <2.0

*n.s* not significant

^a^Pearson Chi square

^b^Unpaired Student’s t test

## Discussion

In accordance with earlier observations [30], the application of NP onto the dorsal nerve roots in rats enhanced the spinal cord nociceptive responses and increased the expression of IL-6, which was used here as an inflammatory marker, in the NP cells. Moreover, the present results demonstrated that miR-223, miR-760 and miR-145 may be up-regulated in NP and released in exosome-like vesicles (ELV) when the NP tissue is exposed to the dorsal nerve roots. In particular, the NP expression of miR-223, which had an anti-nociceptive effect at the spinal level, was increased.

In serum samples from our patients with lumbar radicular pain, the extracellular miR-223 expression was higher when the patients arrived at the clinic than it was 12 months later. Although no clear relationship between miR-223 and IL-6 was observed, a correlation between the change in the miR-223 levels and change in the pain scores was demonstrated. In addition, high levels of miR-223 in the acute phase after disc herniation were associated with a decreased risk of chronic lumbar radicular pain.

These observations suggests that disc herniation also involved changes in miR-223 release. Moreover, herniated discs produce chemotactic factors that promote the recruitment of macrophages and T-cells to the disc [7]. Earlier observations show that miR-223 expressed in the myeloid lineage may be an important modulator of myeloid differentiation and inflammatory responses [33]. In activated macrophages, miR-223 reduces the production of IL-6 and NO [34]. The down-regulation of miR-223, in contrast, has been reported to increase the release of IL-6 and IL-1β [35].

Recent clinical data demonstrate that persistent pain after disc herniation may be associated with low-grade systemic inflammation [10]. An important regulator of inflammatory cytokines is nuclear factor kappa-light-chain-enhancer of activated B cells (NF-κβ). Interestingly, miR-223 can inhibit NF-κβ activation and down-stream signalling including the activation of macrophages/immune cells [34, 36]. In patients with rheumatoid arthritis, miR-223 appears to increase in response to anti-TNF treatment [28]. Moreover, miR-223 may be negatively correlated with inflammatory mediators [28], suggesting that miR-223 could be involved in a negative feedback loop that decreases the cytokines synthesis.

In this process, miR-223, similar to other miRs, can be exchanged between cells via exosomes (Additional file 3). These small vesicles, typically 40–100 nm, may be secreted by several cell-types including NP cells. Earlier data have suggested that exosomes transfer miRs to recipient cells where they repress resident target mRNA translation [20]. Therefore, the up-regulation and release of miR-223 from the NP cells close to the nerve roots could influence on the activity in nearby neuronal tissues, i.e., the DRG or spinal cord. Moreover, previous data show that miR-223 through the NR2B subunits in neuronal tissue may inhibit NMDA induced Ca^2+^ influx [37].

Therefore, miR-223 transferred to recipient cells in the DRG or spinal cord could, as demonstrated in the present study, inhibit nociceptive signalling at the spinal level. Our observation that extracellular miR-223 expression was higher in patients who recovered than those who developed persistent pain, supports this hypothesis. Moreover, profiling studies have demonstrated down-regulation of miRs in the DRG in rats following inflammatory muscle pain [38]. However, there is a debate over how these animal findings translates to patients. Hence, more research is needed to determine the functional role of miR-223 in lumbar radicular pain patients.

## Conclusions

Previous findings show that lumbar radicular pain after disc herniation is associated with up-regulation of inflammatory mediators [9, 10]. The present animal data demonstrated that this process may involve release of small non-coding RNAs including miR-223 in ELVs. An anti-nociceptive effect of miR-223 at the spinal level was also demonstrated. However, increased levels of the miR-223 after disc herniation was only observed in patients who recovered. Hence, dysregulation of miR-223 in the acute phase after disc herniation may be associated with persistent lumbar radicular pain.

## Additional files

**Additional file 1: Table S1.** Additional table.

**Additional file 2: Figure S1.** Additional figure.

**Additional file 3.** Additional datas for Figs 1, 2, 3, 4 and 5.

## Abbreviations

DRG: dorsal root ganglion
ELV: exosome-like vesicles
miR: microRNA
NP: nucleus pulposus
IL-6: interleukin-6
NO: nitric oxide
PGE2: prostaglandin E2
MMP: matrix metalloproteinase
IL-1β: interleukin-1beta
TNF: tumour necrosis factor
VAS: visual analogue scale
NF-κβ: nuclear factor kappa-light-chain-enhancer of activated B cells
ELISA: enzyme-linked immunosorbent assay
rmANOVA: repeated measures analysis of variance
