# Supplementary material for: MicroRNA-223 demonstrated experimentally in exosome-like vesicles is associated with decreased risk of persistent pain after lumbar disc herniation
Source: J Transl Med. 2017 May 1;15:89. doi: 10.1186/s12967-017-1194-8 (PMC5412060; doi:10.1186/s12967-017-1194-8)
Supplement: Supplementary file 1 — Additional file 1: Table S1. Additional table. [file 12967_2017_1194_MOESM1_ESM.docx]

| **Supplementary table 1** |  |  |
| --- | --- | --- |
| **miR-ID** | **Fold Regulation** |  |
| rno-miR-100-5p | -1,2806 |  |
| rno-miR-103-3p | -1,1034 |  |
| rno-miR-107-3p | -1,1314 |  |
| rno-miR-10a-5p | -1,0565 |  |
| rno-miR-124-3p | 5,3543 | A |
| rno-miR-125b-5p | -1,1038 |  |
| rno-miR-127-3p | -1,566 |  |
| rno-miR-128-3p | -1,2591 |  |
| rno-miR-129-2-3p | -1,0092 | B |
| rno-miR-132-5p | 1,5001 | B |
| rno-miR-133a-3p | 1,1421 |  |
| rno-miR-133b-3p | 1,3868 |  |
| rno-miR-134-5p | 1,5247 | B |
| rno-miR-137-3p | -1,0723 |  |
| rno-miR-138-5p | 1,932 |  |
| rno-miR-1-3p | 1,7654 |  |
| rno-miR-142-3p | 6,4271 | * |
| rno-miR-143-3p | 4,3138 |  |
| rno-miR-145-5p | 6,4982 | * |
| rno-miR-146a-5p | 2,3775 |  |
| rno-miR-146b-5p | -2,2382 |  |
| rno-miR-152-3p | -1,067 |  |
| rno-miR-154-5p | 1,8417 |  |
| rno-miR-15b-5p | 1,2947 |  |
| rno-miR-17-5p | 2,0386 |  |
| rno-miR-181a-5p | -1,0001 |  |
| rno-miR-182 | 2,3508 | B |
| rno-miR-183-5p | 11,105 | A |
| rno-miR-199a-3p | -1,1672 |  |
| rno-miR-203a-3p | 1,4132 |  |
| rno-miR-204-3p | 1,9948 |  |
| rno-miR-206-3p | 14,5668 | * |
| rno-miR-208a-3p | 11,9311 | B |
| rno-miR-20a-5p | 2,1437 |  |
| rno-miR-20b-5p | 1,7562 |  |
| rno-miR-210-3p | 1,1008 |  |
| rno-miR-214-3p | 1,1533 |  |
| rno-miR-21-5p | 1,1118 |  |
| rno-miR-219a-2-3p | 1,8645 | A |
| rno-miR-219a-5p | 1,2991 |  |
| rno-miR-223-3p | 9,8618 | * |
| rno-miR-23b-3p | -1,1864 |  |
| rno-miR-29a-3p | -1,1008 |  |
| rno-miR-30a-3p | -1,3455 |  |
| rno-miR-30b-5p | -1,1017 |  |
| rno-miR-30c-5p | -1,189 |  |
| rno-miR-30d-5p | -1,1165 |  |
| rno-miR-31a-5p | -1,0969 |  |
| rno-miR-323-3p | 5,6058 | B |
| rno-miR-324-5p | 1,88 |  |
| rno-miR-325-3p | -5,1857 | B |
| rno-miR-331-5p | -7,1054 | B |
| rno-miR-338-5p | -1,012 |  |
| rno-miR-339-5p | -1,0716 |  |
| rno-miR-34a-5p | -1,1701 |  |
| rno-miR-34c-5p | -1,0597 |  |
| rno-miR-365-3p | -1,1144 |  |
| rno-miR-369-5p | -1,1001 | B |
| rno-miR-374-5p | -1,0991 |  |
| rno-miR-376b-5p | 2,0771 | A |
| rno-miR-378a-3p | 1,4376 |  |
| rno-miR-379-3p | -1,5976 |  |
| rno-miR-384-5p | 2,2862 | B |
| rno-miR-434-3p | 6,3765 | * |
| rno-miR-451-5p | 6,5278 | * |
| rno-miR-463-3p | -3,4032 | A |
| rno-miR-466d |  | C |
| rno-miR-487b-3p | -1,008 |  |
| rno-miR-495 | 2,0786 | B |
| rno-miR-505-5p | 1,2409 |  |
| rno-miR-543-3p | -2,293 | B |
| rno-miR-665 | 3,7547 | B |
| rno-miR-672-3p | -1,1046 |  |
| rno-miR-672-5p | 1,3111 |  |
| rno-miR-674-5p | 1,0293 |  |
| rno-miR-742-3p | -1,3961 |  |
| rno-miR-760-5p | 5,4762 | * |
| rno-miR-7a-5p | -1,066 |  |
| rno-miR-872-5p | 1,0747 |  |
| rno-miR-92a-3p | 1,8321 |  |
| rno-miR-92b-3p | 5,9511 | * |
| rno-miR-96-5p | -3,2212 | B |
| rno-miR-98-5p | -1,0882 |  |
| rno-miR-99a-5p | -1,0964 |  |

* Fold change > 5, follow up with qPCR. A: to high Ct (>30), i.e. low expression in either control or test sample, B: to high Ct (>30), i.e. low expression in both samples, C: non detected.
