# Supplementary figures and images for: MicroRNA-223 demonstrated experimentally in exosome-like vesicles is associated with decreased risk of persistent pain after lumbar disc herniation
Source: J Transl Med. 2017 May 1;15:89. doi: 10.1186/s12967-017-1194-8 (PMC5412060; doi:10.1186/s12967-017-1194-8)

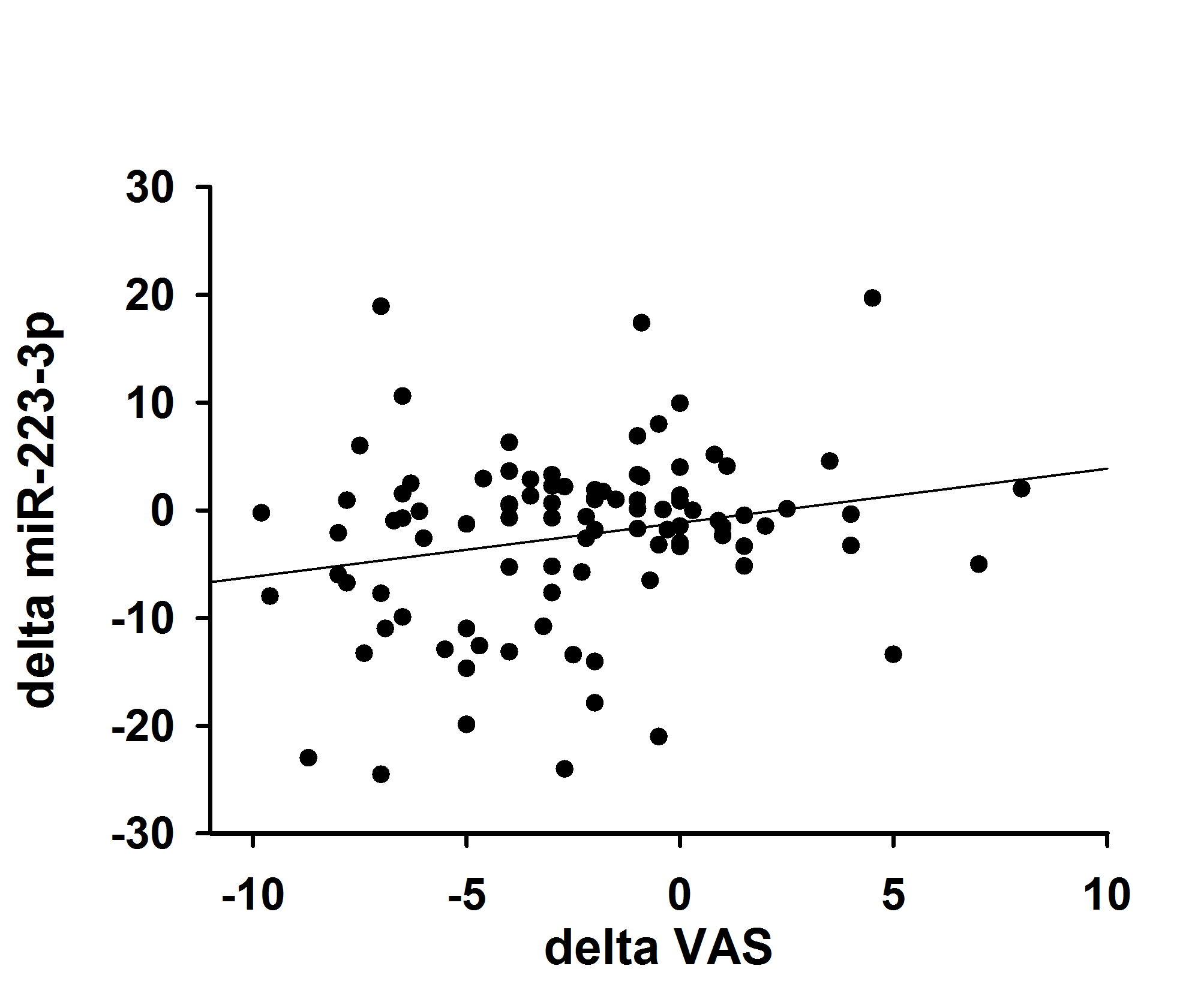

Supplement: Supplementary file 2 — Additional file 2: Figure S1. Additional figure. [file 12967_2017_1194_MOESM2_ESM.png]
